# HIF-2α Regulates NANOG Expression in Human Embryonic Stem Cells following Hypoxia and Reoxygenation through the Interaction with an Oct-Sox Cis Regulatory Element

**DOI:** 10.1371/journal.pone.0108309

**Published:** 2014-10-01

**Authors:** Raffaella Petruzzelli, David R. Christensen, Kate L. Parry, Tilman Sanchez-Elsner, Franchesca D. Houghton

**Affiliations:** Centre for Human Development, Stem Cells and Regeneration, Faculty of Medicine, University of Southampton, Southampton, United Kingdom; University of Dundee, United Kingdom

## Abstract

Low O_2_ tension is beneficial for human embryonic stem cell (hESC) maintenance but the mechanism of regulation is unknown. HIF-2α was found to bind directly to predicted hypoxic response elements (HREs) in the proximal promoter of OCT4, NANOG and SOX2 only in hESCs cultured under hypoxia (5% O_2_). This binding induced an array of histone modifications associated with gene transcription while a heterochromatic state existed at atmospheric O_2_. Interestingly, an enhanced euchromatic state was found when hESCs were exposed to hypoxia followed by 72 hours reoxygenation. This was sustained by HIF-2α which enhanced stemness by binding to an oct-sox *cis*-regulatory element in the NANOG promoter. Thus, these data have uncovered a novel role of HIF-2α as a direct regulator of key transcription factors controlling self-renewal in hESCs but also in the induction of epigenetic modifications ensuring a euchromatic conformation which enhances the regenerative potential of these cells.

## Introduction

hESCs represent a unique source of cells for tissue replacement and regenerative medicine [1], [2]. However, to realize the full potential of hESCs, the delicate balance between self-renewal and early differentiation need to be fully elucidated. Recent studies have found that a physiological O_2_ concentration (2–5%) is beneficial for the maintenance of hESCs in an undifferentiated state [3]–[7].

Maintenance of O_2_ homeostasis is mediated by hypoxia inducible factors (HIFs). These heterodimers are formed of a constitutively expressed HIF-1β (ARNT) subunit and one of the three different HIF-α subunits (HIF-1α, HIF-2α, HIF-3α) [8], [9]. Under normoxia, HIF-α subunits are hydroxylated by prolyl hydroxylase domain enzymes [10], allowing their recognition by the von Hippel Lindau tumor suppressor protein (VHL) initiating HIF-α degradation through the ubiquitin/proteasome complex [11]. In hypoxic conditions, HIFs are stabilised, translocate to the nucleus, bind HIF-1β and induce expression of hypoxia-responsive genes [12]. All three HIF-α subunits bind a canonical recognition sequence (A/G)CGTG termed a hypoxic response element (HRE), in the proximal enhancer or promoter of HIF target genes [13].

In hESCs, HIF-1α was only transiently expressed for approximately 48 h at low oxygen tension [7] while HIF-2α was responsible for the long term response to hypoxia and shown to be an upstream regulator of OCT4 [7], [14], SOX2 and NANOG [7], transcription factors required to maintain self-renewal as well as GLUT1, regulating glucose transport [15]. HIF-2α has also been found to enhance stemness in hESCs following ischemia/reperfusion [16] but the mechanisms which regulate these effects are unknown.

Chromatin organization plays an important role in maintaining self-renewal and pluripotency of hESCs as perturbation of the epigenetic profile of key genes can have profound consequences on stem cell behaviour and fate [17]. Indeed, a high order chromatin structure has been found to regulate OCT4, SOX2 and NANOG in an autoregulatory circuit [18]. Furthermore, a complex genomic interactome might be involved in the regulation of pluripotency-associated genes in order to sustain pluripotency [19]. An improved understanding of the transcriptional regulation of HIFs in hESCs may allow the discovery of a “molecular sensor” that enhances the self-renewal and therapeutic potential of these cells.

In this study, we report that HIF-2α regulates hESC pluripotency by binding directly to the OCT4, NANOG and SOX2 proximal promoters in hESCs cultured at low O_2_ tension. Interestingly, this binding and increased expression was also sustained following 72 hours of reoxygenation leading to activated chromatin structure within the HRE of these transcription factors facilitating an enhanced stemness state. Moreover, ChIP assays revealed that HIF-2α was able to bind an oct-sox *cis*-regulatory element only in the NANOG proximal promoter following reoxygenation. These data suggest that HIF-2α contributes to the formation of a multiprotein complex bringing into physical proximity key pluripotency factors to enhance NANOG expression.

## Materials and Methods

### Cell culture

Hues-7 hESCs (Howard Hughes Medical Institute/Harvard University) [29] were cultured as previously described [15]. Cells were cultured under feeder-free conditions on Matrigel coated plates at either 20% O_2_, or 5% O_2_ for a minimum of 3 passages before use. All cells were removed from the incubator and quickly passaged under atmospheric conditions. Reoxygenation experiments were performed on hESCs cultured at 5% O_2_ for a minimum of 3 passages before being transferred to 20% O_2_ for 72 hours. NT2 cells were maintained in DMEM, 10% fetal bovine serum and 1% penicillin/streptomycin (Invitrogen) at 37°C, 5% CO_2_ and 5% O_2_.

### RT-qPCR

Quantitative Real Time PCR was performed using an ABI 7900 HT Fast Real Time System (Applied Biosystems) in a 384 well plates using a 5 µl reaction containing 1 µg cDNA, 2.5 µl 2x Taqman Universal PCR Master Mix (Applied Biosystems) and 0.25 µl of TaqMan Gene expression Assay Probes (Applied Biosystems) (Table S1 in File S1).

### Immunocytochemistry

hESCs were fixed in 4% paraformaldehyde for 15 min and incubated with 100 mM Glycine for 10 min. Cells were permeabilized in 0.2% Triton-X for 10 min and blocked in 10% fetal calf serum for 30 min before the addition of primary antibodies diluted in 0.6% BSA in PBS. Primary antibodies used were HIF-1α (BD Biosciences) 1∶100 and HIF-2α (Novus Biologicals) 1∶100 and incubated for 90 min. Staining with the secondary antibody was performed with anti-mouse conjugated-FITC (Sigma) 1∶100 or goat anti-rabbit Alexa 488 (Molecular Probes) 1∶700 for 1 hour in 0.6% BSA. Cells were mounted in vectashield containing DAPI (Vecta Laboratories, Peterborough, UK).

### Chromatin Immunoprecipitation assays

ChIP assays were performed as described previously (Forristal et al., 2013) using the following antibodies: HIF-2α (Novus Biologicals), rabbit IgG (Santa Cruz), H3K9me3-Abcam (Abcam), H3K4me3 (Abcam), H3K36 me3 (Abcam), OCT4 (Santa Cruz) and SOX2 (Millipore). Recovered DNA was amplified with custom Taqman Assays (Applied Biosystems) spanning the predicted HRE sites (Tables S2 and S3 in File S1).

### Vector Constructs

A pcDNA3.1 vector containing HIF-2α (EPAS1) (NCBI Reference Sequence: NM_001430.4) gene was kindly provided by Prof. David Russell from the Southwestern Medical Centre (Texas, USA). A region of 630 bp from −437 to +207 bp from the transcription start site of NANOG promoter was amplified with specific primers that contained XhoI and HINDIII restriction sites (NanogXho Forward 5′CTCGAGCGGCTGGTTTCAAACTCCTGA-3′ and NanogHINDIII Reverse 5′-TTCGAACCGGATGCTTCAAAGC-3′). The product was cloned into a TOPO vector (Invitrogen), and removed using XhoI/HINDIII restriction enzymes. The fragment was then cloned into the XhoI/HINDIII sites in a pGL3 Control vector (Promega). This construct was named pGL3-NANOG. Mutagenesis was performed using QuikChange site-directed mutagenesis (Stratagene) following the manufacturer’s procedure and using specific primers (Table S4 in File S1). Luciferase activity was measured using the dual luciferase reporter assay system (Promega) following the manufacturer’s instructions. Normalization was performed using PRL-SV40 Renilla luciferase vector (Promega).

### Transfection and reporter assays

NT2 cells were seeded in 12 well plates (8×10^4^ cells/well) at 5% O_2_ and transiently transfected using SuperFect transfection Reagent (Qiagen) following the manufacturers procedure. pcDNA 3.1 and pGL3 empty vectors were co-transfected to check pcDNA3.1-HEPAS (HIF-2α) activity on the HRE of NANOG proximal promoter harboured by the pGL3-NANOG vector. PRL-SV40 Renilla luciferase vector was used to normalize the transfection efficiency. Luciferase expression was measured using the Luciferase Reporter Assay System (Promega) with a D-20/20 luminometer (Turner Biosystems).

### Statistical analysis

An Anderson-Darling normality test was performed to determine whether data were normally distributed. Relative gene expression differences between cells cultured at 5% and 20% oxygen tension were analysed using a 1-sample *t*-test. Percentage of Input (non-immunoprecipitated chromatin) was calculated as 100×2 ^[Ct (Input)–Ct (IP)]^ for each sample. Differences in chromatin relative enrichment between cells cultured at 5% and 20% oxygen tension were analysed using a Student’s *t*-test. A value of P<0.05 was considered significant. Differences between HIF-2α binding to the oct-sox element or intermediate region were determined using an ANOVA test followed by a Fisher’s test. All data are presented as a mean ± SEM.

## Results

### HIF-2α binds *in* *vivo* to the OCT4, NANOG and SOX2 proximal promoters only in hypoxic conditions in hESCs

**Figure 1:**
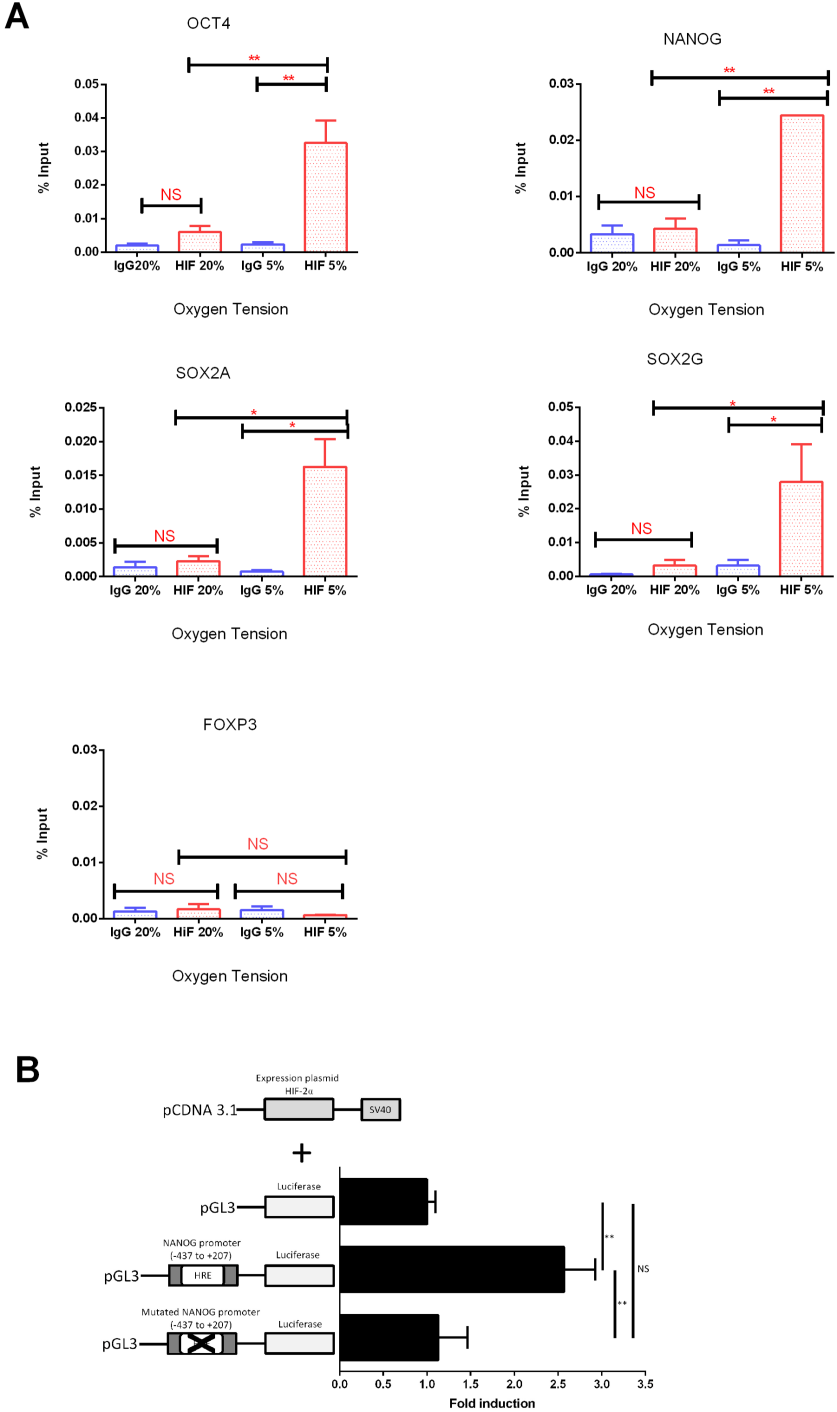
HIF-2α directly binds to HREs in the OCT4, NANOG and SOX2 proximal promoters in hESCs cultured under hypoxia. (A) ChIP analysis of HIF-2α binding a predicted HRE site in the proximal promoters of OCT4, NANOG, SOX2A and SOX2G on chromatin isolated from hESCs cultured either at 20% or 5% O_2_. Amplification of FOXP3 was used as a negative control. DNA enrichment is expressed as a percentage of Input. An average of 3 independent experiments is represented (*P<0.05, **P<0.01; NS: no significant difference). (B) Schematic representation of the HIF-2α expression vector (pcDNA3.1-HIF-2α top panel). The luciferase reporter construct driven by NANOG promoter showed a significant increase (**P<0.01) in luciferase activity compared to the control. Mutation in the predicted HRE caused a significant decrease in the luciferase activity (**P<0.01) compared to the control. An average of 4 independent experiments is shown.

In agreement with Forristal et al. (2010), hESCs cultured at 5% O_2_ tension display an increased expression of OCT4, SOX2 and NANOG compared to those maintained at 20% O_2_ (Figure S1 in File S1). To verify whether endogenous HIF-2α interacts with the predicted HREs in the promoter region of OCT4, NANOG and SOX2 genes, ChIP analysis was performed on hESCs cultured either at 5% or 20% O_2_. Amplification of a putative HRE in both the OCT4 and NANOG promoter revealed a 4-fold enrichment (P<0.01) in cells cultured at 5% O_2_ compared to the IgG control. In contrast, hESCs cultured at 20% O_2_ revealed no significant enrichment compared to the IgG control (Fig. 1A). For the SOX2 proximal promoter, 2 different HREs situated at −1450 bp and −1100 bp from the transcriptional start site were analysed. These HREs differ by the presence of the A nucleotide (A)CGTG (SOX2A) or a G nucleotide (G)CGTG (SOX2G). ChIP samples showed a 6 fold (P<0.05) and 3 fold (P<0.05) increase in the level of HIF-2α binding in the promoter of SOX2A and SOX2G respectively compared to the IgG control (Fig. 1A). To further verify the specificity of HIF-2α binding, a negative control probe specific for the FOXP3 promoter was used. This probe was designed to amplify a region in the proximal promoter which does not contain an HRE but instead was situated between 2 potential HREs at −670 bp and +104 bp from the transcription start site (Fig. 1A). qPCR on three independent ChIP experiments on chromatin derived from hESCs cultured at either 5% or 20% O_2_ revealed no significant enrichment for HIF-2α in this FOXP3 promoter region (P>0.05). These data indicate that HIF-2α binds directly to HREs in the proximal promoter of OCT4, NANOG and SOX2 only in hypoxic conditions in hESCs.

### Functional Analysis of the HRE in the NANOG proximal promoter

To verify the novel, direct binding of endogenous HIF-2α in the HRE (−301 bp) to the NANOG proximal promoter and to interrogate whether this was functional and able to activate transcription, luciferase reporter assays were performed in NT2 cells. By cloning a fragment of the proximal NANOG promoter (−437 bp to +207 bp from the transcription start site), that includes the HRE site for HIF-2α (pGL3-NANOG −301 bp) into a PGL3 vector, a significant promoter-driven increase in transcription was observed (P<0.001) only when NT2 cells were co-transfected with a HIF-2α expression vector (Fig. 1B). Furthermore, a significant reduction (P<0.001) of NANOG transcription was observed when a mutant Renilla plasmid vector (pGL3-Mut NANOG −301 bp), where the HRE sequence had been abrogated, was co-transfected with HIF-2α, when compared to the wild type vector (Fig. 1B). This confirms that HIF-2α directly binds the HRE at −301 bp from the start site in the NANOG promoter and is functionally responsible for NANOG transcription.

### Reoxygenation affects the mRNA profile of genes regulating self-renewal

**Figure 2:**
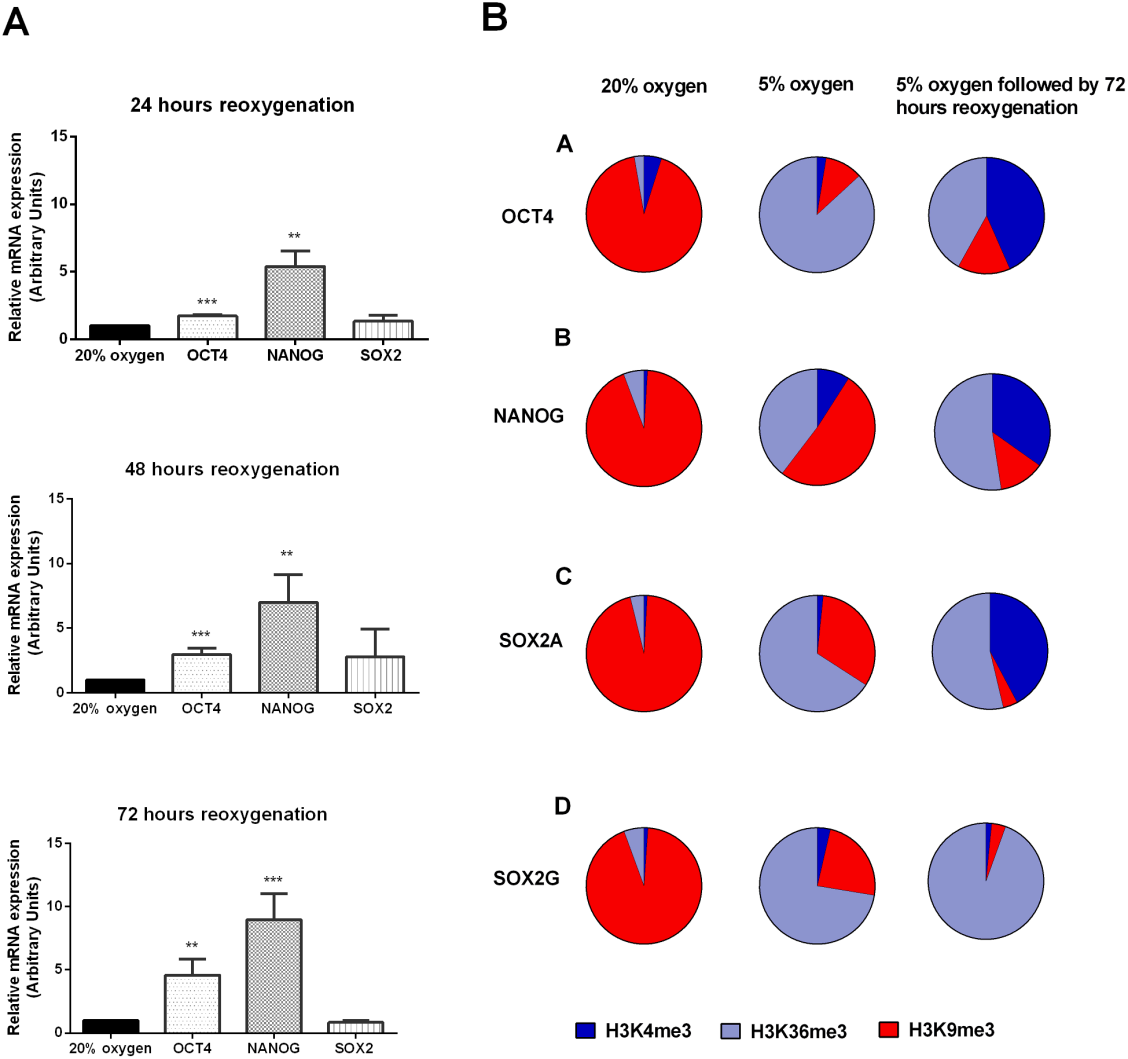
Hypoxia and reoxygenation enhance the expression of pluripotency genes through a euchromatic state within the HRE site. (A) RT-qPCR analysis for OCT4, NANOG and SOX2 mRNA in hESCs subjected to 24, 48 and 72 hours reoxygenation compared to those maintained at 20% O_2_. All data have been normalized to UBC and to 1 for hESCs maintained at 20% O_2_. Values are mean of 5 independent experiments ± SEM (*P<0.05, **P<0.01, ***P<0.001 significantly different from 20% O_2_). (B) Pie charts showing ChIP analysis of histone modification markers H3K4me3, H3K36me3 and H3K9me3 binding a predicted HRE site in the proximal promoters of OCT4, NANOG, SOX2A and SOX2G in hESCs exposed to 20% O_2_, 5% O_2_, and hypoxia reoxygenation respectively. DNA enrichment is expressed as a percentage of Input relative to the IgG control. An average of 3 to 4 independent experiments is represented.

We next investigated whether reoxygenation following hypoxia affects OCT4, SOX2 and NANOG expression in hESCs. Using RT-qPCR, NANOG expression was significantly increased after 24 hours of reoxygenation and remained highly expressed following exposure to reoxygenation for 72 hours (Fig. 2A). Similarly, OCT4 mRNA showed a more gradual but significant increase in expression between 24–72 hours of reoxygenation. In contrast, there was no difference in SOX2 mRNA expression upon reoxygenation. These data were intriguing and suggested that reoxygenation could also have an influence on the epigenetic state of hESCs.

### Hypoxia alters the histone modification profile in the proximal promoter of key transcription factors regulating hESC self-renewal

To determine the impact of hypoxia on chromatin modifications around the predicted HRE site in OCT4, NANOG and SOX2 proximal promoters, ChIP assays using H3K4me3 and H3K36me3, markers of transcriptional activation, and H3K9me3 a marker of gene silencing were performed. Pie charts were used to represent the percentage input precipitated by each modified histone as a proportion of the total, for each gene of interest whereas bar charts were used to show comparison of individual epigenetic marks and are shown in the Supplementary data. Using probes designed to cover the HRE site at −1956 bp in the OCT4 proximal promoter, a significant proportion of the H3K36me3 histone marker was found in hESCs cultured under hypoxic conditions compared to 20% O_2_ (Fig. 2B and Figure S2 in File S1). A similar trend of histone modification levels were obtained when the HRE at −301 bp in the NANOG proximal promoter and the HREs at −1450 bp and −1100 bp from the start site in the SOX2 proximal promoter were amplified in hESCs cultured under hypoxic conditions (Fig. 2B and Figure S2 in File S1). hESCs maintained at 20% O_2_ displayed high H3K9me3 levels and significantly reduced H3K4me3 and H3K36me3 within the NANOG and SOX2 HREs compared to cells cultured under hypoxic conditions. These data confirm that the chromatin state in hESCs cultured at 20% O_2_ tension is more heterochromatic and inaccessible to transcription factors or chromatin remodelling factor binding (Fig. 2B and Figure S2 in File S1). Overall, at 5% O_2_ a significant increase of the H3K36me3 levels was observed for OCT4, SOX2 and NANOG. These trends are consistent with a more open conformational chromatin under low levels of O_2_ and indicate active changes that correlate with transcriptional activity.

### Hypoxia/reoxygenation induces a euchromatic state within the HREs of OCT4, SOX2 and NANOG proximal promoters

To investigate whether the epigenetic profile within the HRE site was affected following reoxygenation, ChIP assays were performed on hESCs subjected to hypoxia followed by 72 hours of reoxygenation. Surprisingly, an overall increased proportion of H3K4me3 and H3K36me3 and a dramatic reduction in the proportion of H3K9me3 bound was found within the HRE in OCT4, SOX2A promoter compared with chromatin of hESCs cultured at 20% O_2_ (Fig. 2B and Figure S2 in File S1). Interestingly, these differences were particularly marked for the HRE within the NANOG promoter (Fig. 2B and Figure S2 in File S1) suggesting that, when cells were exposed to hypoxia/reoxygenation chromatin retains a more open conformational state, compared to normoxic conditions (Fig. 2B).

### HIF-2α directly interacts with the HRE of pluripotency genes after 3 days reoxygenation

**Figure 3:**
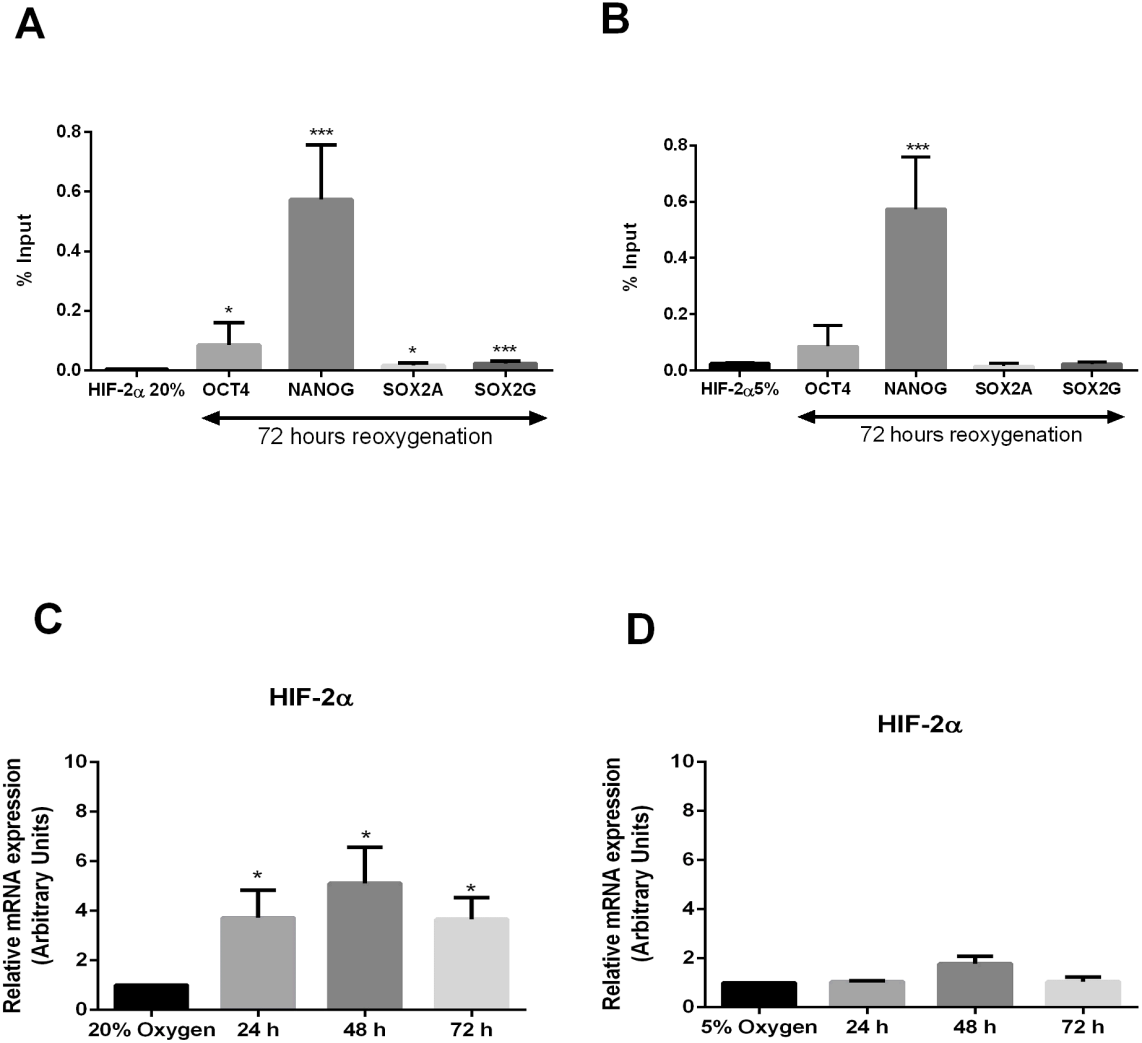
HIF-2α sustains the expression of NANOG following hypoxia/reoxygenation. ChIP analysis of HIF-2α binding a predicted HRE site in the proximal promoters of OCT4, NANOG, SOX2A and SOX2G on chromatin isolated from hESCs subjected to hypoxia followed by 72 hours reoxygenation. HIF-2α binding to the HRE of pluripotency genes is expressed relative to 20% O_2_ (A) or 5% O_2_ (B). DNA enrichment is expressed as a percentage of Input. An average of 4 independent experiments is represented (*P<0.05, ***P<0.001). RT-qPCR for HIF-2α expression in hESCs subjected to 24, 48 and 72 hours reoxygenation. All data have been normalized to UBC and to 1 for hESCs cultured at 20% O_2_ (C) or 5% O_2_ (D). Values are mean of 3 to 4 independent experiments ± SEM (*P<0.05).

We next considered that nuclear HIF-2α may not be degraded following reoxygenation but instead may bind to the HRE of all core pluripotency genes and could be responsible for these epigenetic changes. ChIP assays were performed on hESCs collected after 72 hours reoxygenation. HIF-2α showed a significant enrichment in the HRE within the OCT4 (P<0.04), NANOG (P<0.001), SOX2A (P<0.05) and SOX2G (P<0.001) promoter compared with chromatin isolated from hESCs cultured at 20% O_2_ (Fig. 3A). Thus, our data indicates that at least nuclear HIF-2α protein is stabilised and not degraded following hypoxia and 72 hours of reoxygenation as was further confirmed using immunocytochemistry (Figure S3 in File S1). Interestingly, when compared to hESCs maintained at 5% O_2_, there was a significant enrichment of HIF-2α binding only to the NANOG promoter (P<0.01) (Fig. 3B). These data reveal that HIF-2α is able to sustain higher NANOG transcriptional activation together with moderate levels of OCT4 and SOX2 after an oxidative insult. Moreover, HIF-2α mRNA expression was found to be significantly higher on each day post-reoxygenation compared to cells cultured at 20% O_2_ (Fig. 3C). Interestingly, this increased HIF-2α expression was similar to that observed in cells cultured under hypoxic conditions (Fig. 3D). These data suggest that HIF-2α is transcriptionally regulated and sustains high nuclear protein levels following ischaemic preconditioning.

We also wanted to investigate the effect of hypoxia following 72 hours of reoxygenation on HIF-1α using immunocytochemistry. As expected, HIF-1α protein was present in hESCs after 24 hours of hypoxia but was absent in cells maintained for at least 3 passages at 5% O_2_ (Figure S4 in File S1) [7]. HIF-1α was also not expressed in hESCs following hypoxia and 72 hours of reoxygenation (Figure S4 in File S1). This confirms that HIF-1α sustains only the first adaptation to hypoxia and highlights the unique role of HIF-2α in regulating hESC self-renewal not only under hypoxia but also following reoxygenation.

### HIF-2α binds to an oct-sox *cis* regulatory element within the NANOG promoter

**Figure 4:**
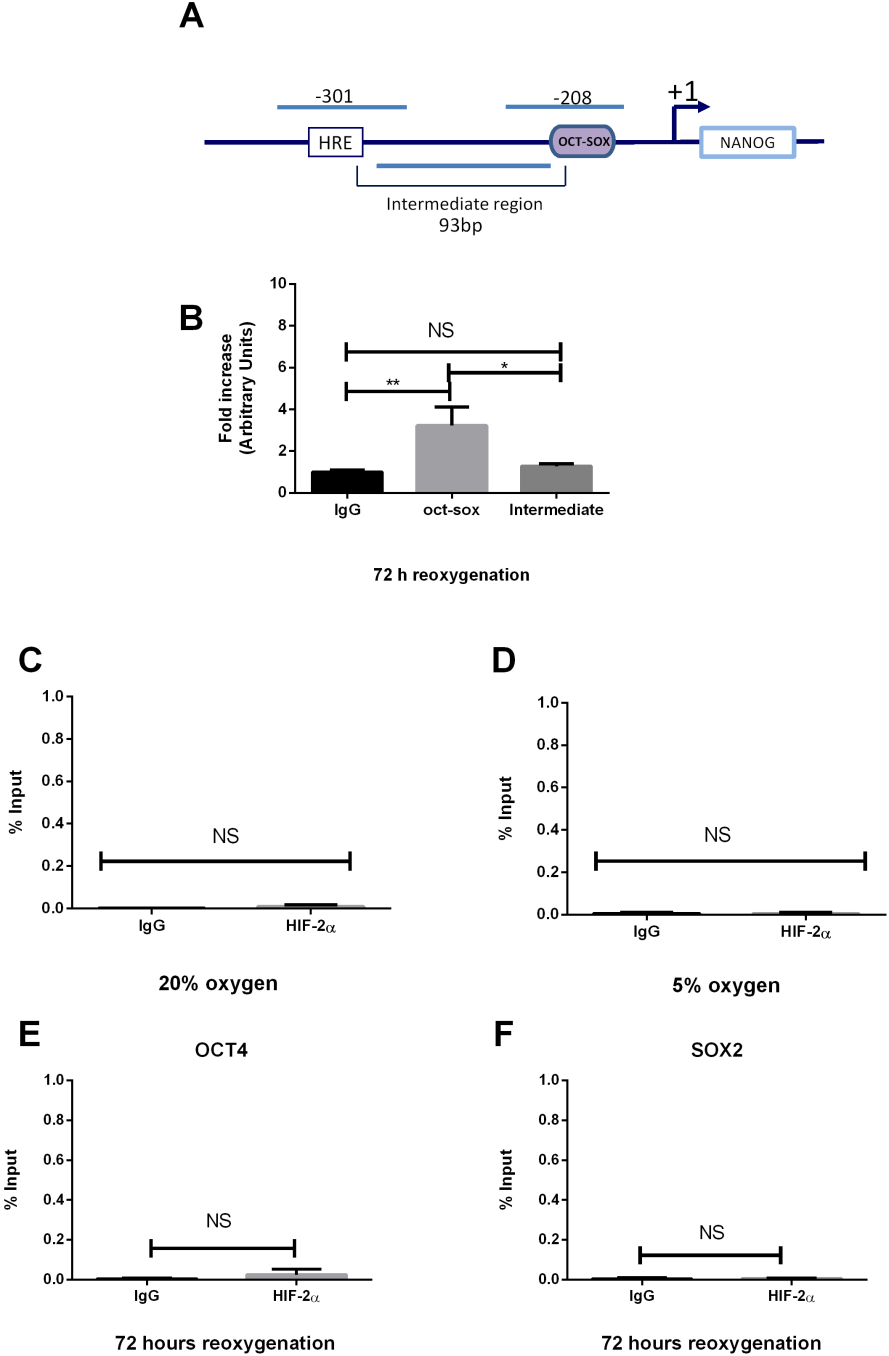
Hypoxia/reoxygenation induces HIF-2α binding to the oct-sox *cis* element in the NANOG promoter. (A) Schematic representation of the probes designed to cover the HRE, oct-sox *cis* element and intermediate region on the NANOG proximal promoter. (B) ChIP analysis of HIF-2α binding to the oct-sox-*cis* element and the intermediate region in hESCs cultured under hypoxia followed by 72 hours reoxygenation. An average of 4 independent experiments is represented (*P<0.05, **P<0.01). ChIP analysis of HIF-2α binding the oct-sox *cis*-regulatory element in NANOG proximal promoter on chromatin isolated from hESCs cultured at 5% O_2_ (C), 20% O_2_ (D). ChIP analysis of HIF-2α binding the oct-sox *cis*-regulatory element in OCT4 proximal promoter (E) and SOX2 intron (F) on chromatin isolated from hESCs subjected to 72 hours reoxygenation. DNA enrichment is expressed as a percentage of Input. An average of 3 independent experiments is represented. (NS: no significant difference).

Bioinformatic analysis of the NANOG promoter revealed an oct-sox *cis*-regulatory element at −208 bp (TTTGCATTACAATG) from the transcription start site and in close proximity (93 bp) to the HRE (Fig. 4A). We considered the possibility that HIF-2α could be responsible for a three-dimensional chromatin structure that keeps transcription factors more closely together when cells are cultured at either 5% O_2_, 20% O_2_ or following hypoxia/reoxygenation. Using ChIP assays, HIF-2α was found to bind to the oct-sox element of the NANOG promoter only when hESCs were subjected to hypoxia followed by 72 hours reoxygenation (Fig. 4B). In contrast, no binding to this element was observed in hESCs cultured at either 5% (Fig. 4C), or 20% O_2_ (Fig. 4D). Moreover, no interaction was found within the oct-sox element in the OCT4 promoter (Fig. 4E) and SOX2 intron (Fig. 4F) in hESCs subjected to hypoxia followed by 72 hours reoxygenation. The observed interaction of HIF-2α and the oct-sox element could be experimentally dependent on the fact that both HRE and oct-sox are in the same DNA molecule and relatively close (93 bp), which could potentially lead to both sites precipitating together as an artifact. To rule this possibility out, we designed a control probe that covers the 93 bp sequence between the HRE site and the oct-sox element in the NANOG promoter (called intermediate region) and expected this region not to be present in the immunoprecipitated samples (Fig. 4A, B). qPCR on four independent ChIP experiments on chromatin derived from hESCs subjected to hypoxia and reoxygenation for 72 hours and immunoprecipitated with HIF-2α revealed no significant binding to the intermediate region compared to the oct-sox element and the IgG control. (Fig. 4B). These results show that both the HRE and oct/sox binding sites are in physical contact with HIF-2α only when hESCs are exposed to reoxygenation. This suggests that HIF-2α functions as an enhancer to increase the expression of NANOG under conditions of reoxygenation.

### Functional Analysis of the oct-sox element in the NANOG proximal promoter

**Figure 5:**
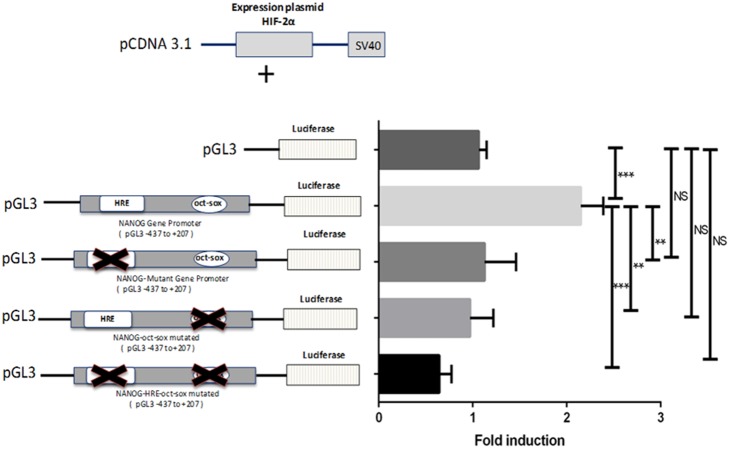
HIF-2α binds the oct-sox *cis*-regulatory element and drives NANOG activity. Schematic representation of the HIF-2α expression vector (pcDNA3.1-HIF-2α top panel). A pcDNA3.1-HIF-2α vector was transiently co-transfected in NT2 cells with a luciferase reporter construct driven by NANOG promoter with intact HRE and oct-sox element or with different constructs in which either the HRE, the oct-sox or both sites were mutated. Luciferase activity was significantly decreased when the HRE (P<0.01), the oct-sox element (P<0.01) and both sites (P<0.001) were mutated compared to the NANOG gene promoter construct with the unmutated HRE and oct-sox element. An average of 4 independent experiments is shown.

To verify the functionality of the HIF-2α binding to the oct-sox cis regulatory element (−208 bp) in the NANOG proximal promoter, Luciferase reporter assays were performed in NT2 cells. A 630 bp genomic sequence of the NANOG promoter which contains the HRE site for HIF-2α (−301 bp) and the oct-sox *cis* regulatory element (−208 bp) was cloned into a PGL3 vector. In addition, two Renilla plasmid vectors were used where the oct-sox *cis* element (pGL3-Mut NANOG −208 bp) or both the HRE and the oct-sox elements (PGL3-Mut NANOG −301/Mut −208 bp) were mutated in order to disrupt the HIF-2α binding. All plasmids were co-transfected with a HIF-2α expression vector (pcDNA3.1-HIF-2α) in NT2 cells. Luciferase reporter assays showed a significant reduction of the promoter-driven transcription (P<0.05) when NT2 cells were co-transfected with a HIF-2α expression vector and a mutant of the HRE site (pGL3-Mut NANOG −301 bp) compared to the wild type sequence (pGL3-NANOG −301 bp). Upon mutation of the oct-sox element within the NANOG promoter (pGL3-Mut NANOG −208 bp), the level of activation decreased to about half (P<0.01) of that for wild type NANOG (Fig. 5). Interestingly, when both the HRE and oct-sox elements were mutated in NANOG (PGL3-Mut NANOG −301/Mut −208 bp), there was an even greater reduction in luciferase activity (P<0.001; Fig. 5) suggesting that HIF-2α is able to interact not only with the HRE sequence but also with the oct-sox element in the NANOG proximal promoter and that this binding is functionally responsible for NANOG activity.

### OCT4 and SOX2 bind to the oct-sox element within the NANOG promoter in normoxia, hypoxia and following 72 hours of reoxygenation

**Figure 6:**
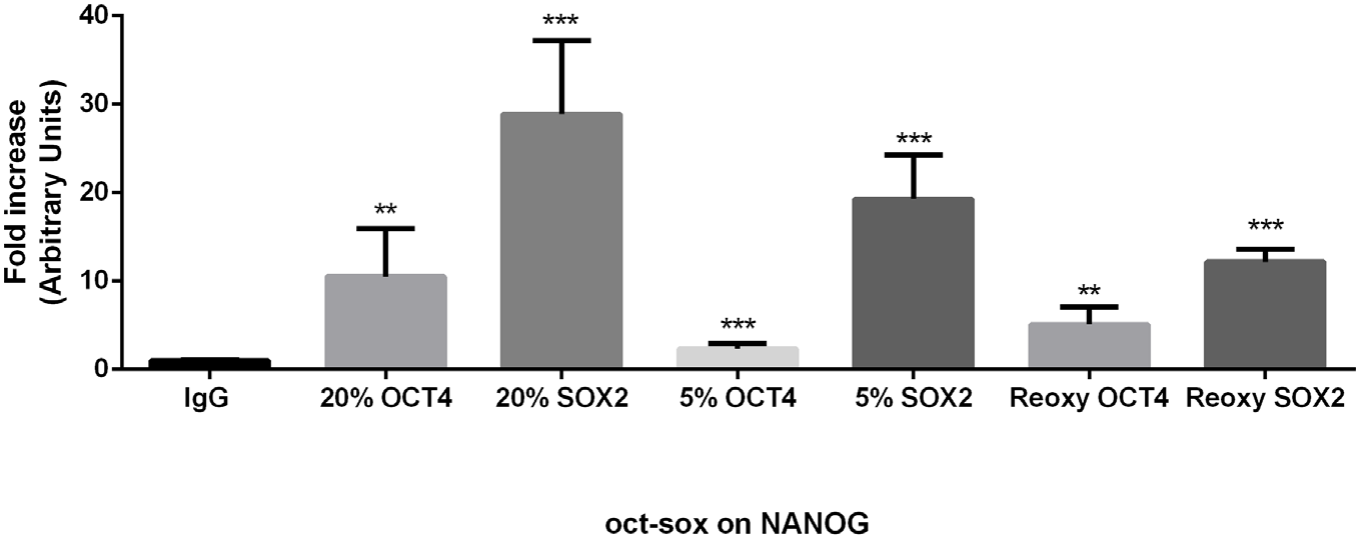
OCT4 and SOX2 bind to the oct-sox *cis* element in the NANOG proximal promoter. ChIP analysis of OCT4 and SOX2 binding to the oct-sox *cis* element in hESCs cultured at either 5% O_2_, 20% O_2_, or 5% O_2_ followed by 72 hours reoxygenation. An average of 4 independent experiments is represented (**P<0.01, ***P<0.001).

Given that OCT4 and SOX2 play a key role in controlling hESC pluripotency through the transcriptional regulation of NANOG [20], we next considered the effect of oxygen tension in the binding of OCT4 and SOX2 to the composite oct-sox element within the NANOG promoter. ChIP assays were carried out with OCT4 and SOX2 antibodies in hESCs cultured under normoxia, hypoxia and following 72 hours of reoxygenation. The ChIP material was amplified by qPCR using a specific probe which covers the oct-sox element within the NANOG proximal promoter (−208 bp). ChIP assays showed a significant 15 fold (P<0.01) and 35 fold (P<0.001) increase of OCT4 and SOX2 binding within the oct-sox *cis* regulatory element in the NANOG proximal promoter when hESCs were maintained at 20% O_2_ (Fig. 6). Interestingly, when hESCs were cultured at 5% O_2_, OCT4 and SOX2 binding to the oct-sox element dropped to 2 fold (P<0.001) and 20 fold (P<0.001) respectively compared to the IgG control while the level of enrichment decreased to about 5 fold (P<0.01) and 10 fold (P<0.001) when hESCs were exposed to hypoxia followed by reoxygenation (Fig. 6). These data confirm the occupancy of OCT4 and SOX2 at the oct-sox element within the NANOG promoter in undifferentiated hESCs in all the oxygen conditions tested. Furthermore, these results suggest that under normoxia NANOG transcription is mainly influenced by OCT4 and SOX2, while under hypoxia and particularly following reoxygenation, there is a trend towards reduced binding of these proteins.

## Discussion

Accumulating evidence suggests that resident stem/progenitor cell populations are able to promote survival in response to oxidative stress and repopulate damaged tissues. However the precise “stemness” feature which promotes stem cell survival and regeneration is not fully understood. This study proposes that hESCs exposed to hypoxia and reoxygenation maintain their stemness through a *novel* mechanism of epigenetic regulation whereby HIF-2α enhances NANOG expression to protect cells from oxidative stress. Our data provide evidence that endogenous HIF-2α binds directly to HREs in the proximal promoter of OCT4, NANOG and SOX2 in hESCs cultured under hypoxia but not at atmospheric O_2_ tensions. Moreover, a direct involvement of HIF-2α in NANOG gene expression was observed not only under hypoxia but also following reoxygenation. As NANOG has a central role in hESC pluripotency and self-renewal, but also in cell cycle progression [21]–[23] HIF-2α binding could explain the increased rate of proliferation observed in hESCs under hypoxia [7]. Our ChIP data showed that HIF-2α preferentially binds to SOX2A compared to the SOX2G. The biological significance of this result is not known but may reflect the presence of other transcription factors that can cooperate with HIF-2α to regulate SOX2 expression, or the difference observed in the chromatin conformation around the HRE.

Consistent with these data, a heterochromatic state was observed under atmospheric O_2_ tensions. Since HIF-2α is not degraded under these conditions but displays a cytoplasmic localization [7], the high levels of H3K9me3 and absence of activation markers H3K4me3 and H3K36me3 at the HRE are likely to explain the lack of binding to OCT4, SOX2 and NANOG. In contrast, a bivalent chromatin state was observed under hypoxic conditions in the HRE of pluripotency genes with moderate levels of H3K9me3 which likely reflect the hypoxic induction of G9a methyltransferase [24] and increased levels of H3K36me3 which is associated with gene transcription elongation [25]. Hence, hESCs may respond rapidly to O_2_ changes by accumulating OCT4, NANOG and SOX2 transcripts. Surprisingly, only a small increase in H3K4me3 was observed in the predicted HRE site under hypoxic conditions and may be due to a family of jumonji-domain histone demethylases such as JARID1B, known to be regulated by HIF-1α and HIF-2α [26].

In order to mimic the effect of an ischemia-reperfusion environment, we subjected hESCs to reoxygenation. Histone modification analysis showed that H3K4me3 and H3K36me3 levels remained high while H3K9me3 was dramatically reduced compared to hESCs cultured at 5% O_2_. These modifications were associated with a direct interaction of HIF-2α within the HRE of all core pluripotency genes but particularly with the NANOG gene promoter. This finding was intriguing since HIF-2α was not degraded following reoxygenation suggesting either a different mechanism of degradation and/or post-transcriptional modification. Recently, low p53 and high HIF-2α levels were also found to be associated with increased NANOG expression upon reoxygenation [16]. Our data extends this finding and suggests that epigenetic modifications associated with HIF-2α binding may promote NANOG activity, a mechanism which likely protects cells from oxidative stress.

**Figure 7:**
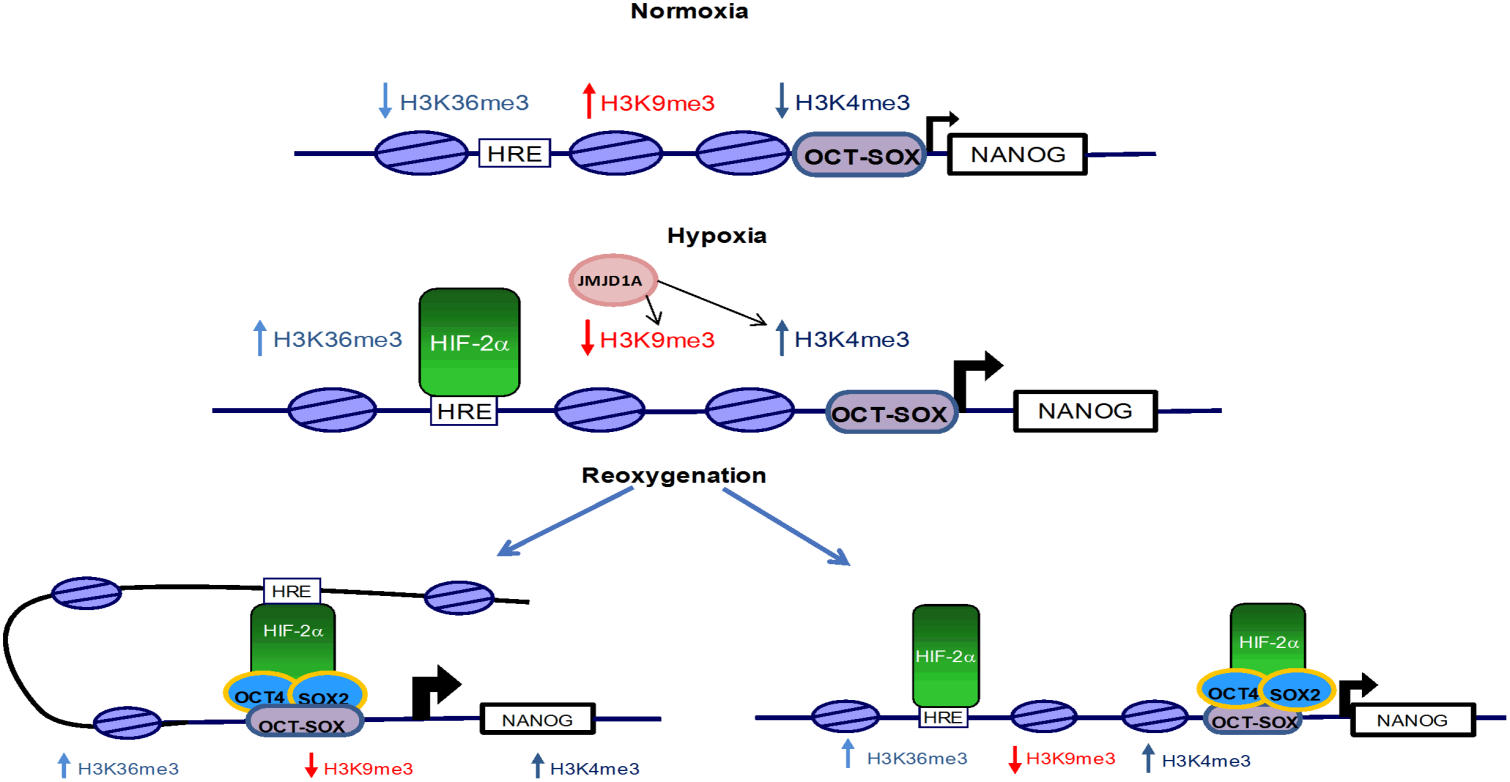
Schematic representation of NANOG promoter regulation under conditions of normoxia, hypoxia and reoxygenation in hESCs.

We identified a novel, specific interaction between HIF-2α and an oct-sox *cis* regulatory element in the NANOG promoter. This interaction occurred only when hESCs were subjected to hypoxia followed by reoxygenation and might be the result of the formation of a tight chromatin structure on the NANOG promoter, bringing together the HRE and oct-sox site through HIF-2α and other ancillary proteins thereby leading to an increased expression (Fig. 7 bottom left). Another possibility that cannot be ruled out is that reoxygenation generates a new binding site for HIF-2α that then binds to the oct-sox element, independently of the binding to the HRE site (Fig. 7 bottom right). These will need further investigation however it is tantalizing to hypothesize that a tight loop conformation could form a protective “pocket” that may shield HIF-2α from degradation, maintaining its nuclear localization upon reoxygenation. Surprisingly, this binding was not present under hypoxic or normoxic conditions alone.

The oct-sox element is functionally important for NANOG expression and establishing a pluripotent phenotype in stem cells [27] but our data highlight that OCT4 and SOX2 are not the only proteins that can bind and allow the pluripotent-specific expression of NANOG. Hence, regardless of the molecular structure of the promoter, our data suggest that, under reoxygenation, hESCs activate an alternative mechanism of regulation in which HIF-2α activity is central to the maintenance of self-renewal, through the interaction with an oct-sox element. Indeed, binding of HIF-2α to the oct-sox element is functionally active and able to confer NANOG transcriptional activation since disruption of the oct-sox element or both HRE and oct-sox sequences are able to reduce NANOG activity. Furthermore, we found that OCT4 and SOX2 binding to the oct-sox element was particularly enriched when hESCs were cultured at 20% O_2_ while less binding occurred in hypoxia and following 72 hours of reoxygenation. These data allow us to speculate that the presence of HIF-2α in hypoxia and reoxygenation takes over the activation of NANOG in hESCs possibly working in combination with OCT4 and SOX2 which simultaneously associate with the oct-sox element. We also observed that SOX2 binding was more enriched within the NANOG oct-sox element compared to OCT4. This will need further investigation but since SOX2 is known to bend DNA [28], this transcription factor may in part contribute to the chromatin conformation within the oct-sox element that leads to recruitment of HIF-2α in hypoxia and reoxygenation.

In conclusion, we demonstrate that HIF-2α is recruited as a co-activator to the oct-sox element under reoxygenation, subsequently enhancing NANOG expression. We therefore propose a novel mechanism of epigenetic regulation in hESCs whereby HIF-2α binds the HRE and forms a multiprotein complex together with OCT4 and SOX2 and other chromatin remodelling factors in order to enhance NANOG expression following hypoxia/reoxygenation to protect cells from oxidative stress. This epigenetic mechanism may be part of a molecular program, characteristic of resident stem/progenitor cell populations that repopulate and promote survival during post-injury stress, or in a wide range of diseases associated with ischemia and reoxygenation.

## Supporting Information

File S1 **Supporting information.** Figure S1, Pluripotency markers are reduced in hESCs cultured at 20% O_2_ condition compared to hES cells cultured at 5% O_2_. RT-qPCR analysis of OCT4, SOX2 and NANOG expression in hESCs cultured at 5% or 20% O_2_. All data have been normalized to UBC and to 1 for 5% O_2_. Values are mean of 4 independent experiments ± SEM (*P<0.05). Figure S2, Histone modifications induced within the HRE of OCT4, NANOG and SOX2 genes in hESCs cultured in hypoxia and following reoxygenation. ChIP assays H3K4me3, H3K9me3 or H3K36me3 on chromatin isolated from hESCs cultured either at 20% O_2_, 5% O_2_ or 72 hours post-reoxygenation. Data have been normalized to 5% O_2_ for hESCs cultured at 20% O_2_ (A), or either to 20% O_2_ (B) or 5% O_2_ (C) for hESCs subjected to reoxygenation. DNA enrichment is expressed as a percentage of input minus the background IgG. An average of 3 to 4 independent experiments is represented (*P<0.05, **P<0.01; ***P<0.001). Figure S3, Expression of HIF-2α in hESCs cultured at 5% O_2_ followed by 72 hours of reoxygenation (Reoxy). Protein expression of HIF-2α (A and B), merged with DAPI (B) and the secondary antibody only negative control (C and D), merged with DAPI (D) of hESCs cultured on Matrigel under hypoxic conditions followed by 72 hours of reoxygenation. Scale bar 25 µm. Figure S4, Expression of HIF-1α in hESCs cultured at 5% O_2_ for at least 3 passages (5% O_2_), 5% O_2_ followed by 72 h of reoxygenation (Reoxy), or 20% O_2_ followed by 24 hours at 5% O_2_ (24 h 5% O_2_). Protein expression of HIF-1α (A–E), merged with DAPI (B, D, F) and the secondary antibody only negative control (G, H), merged with DAPI (H) of hESCs culture at 5% O_2_ for at least 3 passages (A–B), 5% O_2_ followed by 72 hours reoxygenation (C–D), or 20% O_2_ followed by 24 hours at 5% O_2_ (E–F). Scale bar 25 µm. Table S1, Table S2, Table S3, Table S4.
(DOCX)
