# Supplementary material for: HIF-2α Regulates NANOG Expression in Human Embryonic Stem Cells following Hypoxia and Reoxygenation through the Interaction with an Oct-Sox Cis Regulatory Element
Source: PLoS One. 2014 Oct 1;9(10):e108309. doi: 10.1371/journal.pone.0108309 (PMC4182711; doi:10.1371/journal.pone.0108309)
Supplement: File S1 — Supporting information. Figure S1, Pluripotency markers are reduced in hESCs cultured at 20% O2 condition compared to hES cells cultured at 5% O2. RT-qPCR analysis of OCT4, SOX2 and NANOG expression in hESCs cultured at 5% or 20% O2. All data have been normalized to UBC and to 1 for 5% O2. Values are mean of 4 independent experiments ± SEM (*P<0.05). Figure S2, Histone modifications induced within the HRE of OCT4, NANOG and SOX2 genes in hESCs cultured in hypoxia and following reoxygenation. ChIP assays H3K4me3, H3K9me3 or H3K36me3 on chromatin isolated from hESCs cultured either at 20% O2, 5% O2 or 72 hours post-reoxygenation. Data have been normalized to 5% O2 for hESCs cultured at 20% O2 (A), or either to 20% O2 (B) or 5% O2 (C) for hESCs subjected to reoxygenation. DNA enrichment is expressed as a percentage of input minus the background IgG. An average of 3 to 4 independent experiments is represented (*P<0.05, **P<0.01; ***P<0.001). Figure S3, Expression of HIF-2α in hESCs cultured at 5% O2 followed by 72 hours of reoxygenation (Reoxy). Protein expression of HIF-2α (A and B), merged with DAPI (B) and the secondary antibody only negative control (C and D), merged with DAPI (D) of hESCs cultured on Matrigel under hypoxic conditions followed by 72 hours of reoxygenation. Scale bar 25 µm. Figure S4, Expression of HIF-1α in hESCs cultured at 5% O2 for at least 3 passages (5% O2), 5% O2 followed by 72 h of reoxygenation (Reoxy), or 20% O2 followed by 24 hours at 5% O2 (24 h 5% O2). Protein expression of HIF-1α (A–E), merged with DAPI (B, D, F) and the secondary antibody only negative control (G, H), merged with DAPI (H) of hESCs culture at 5% O2 for at least 3 passages (A–B), 5% O2 followed by 72 hours reoxygenation (C–D), or 20% O2 followed by 24 hours at 5% O2 (E–F). Scale bar 25 µm. Table S1, Table S2, Table S3, Table S4. (DOCX) [file pone.0108309.s001.docx]

**Supporting Information**

**HIF-2α regulates NANOG expression in human embryonic stem cells following hypoxia and reoxygenation through the interaction with an oct-sox cis regulatory element**

Raffaella Petruzzelli, David R. Christensen, Kate L. Parry, Tilman Sanchez-Elsner and Franchesca D. Houghton


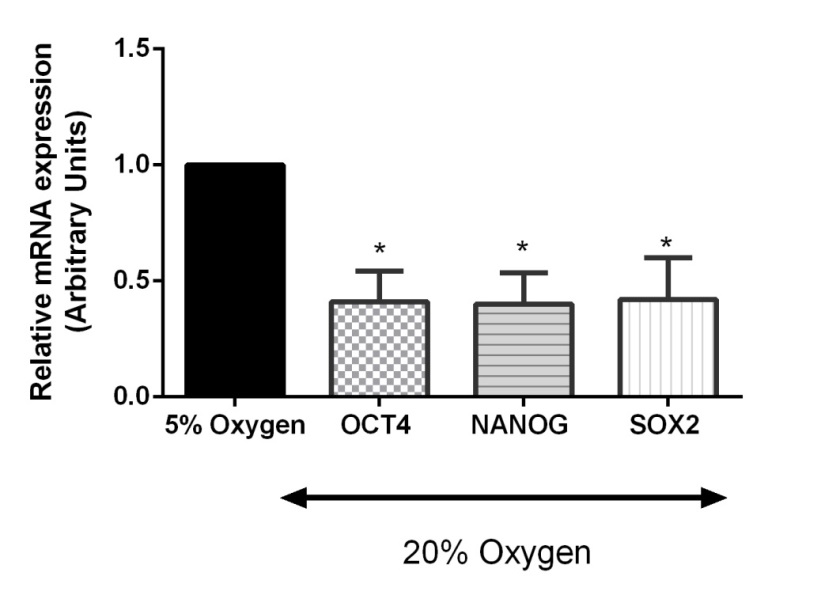


**Figure S1**


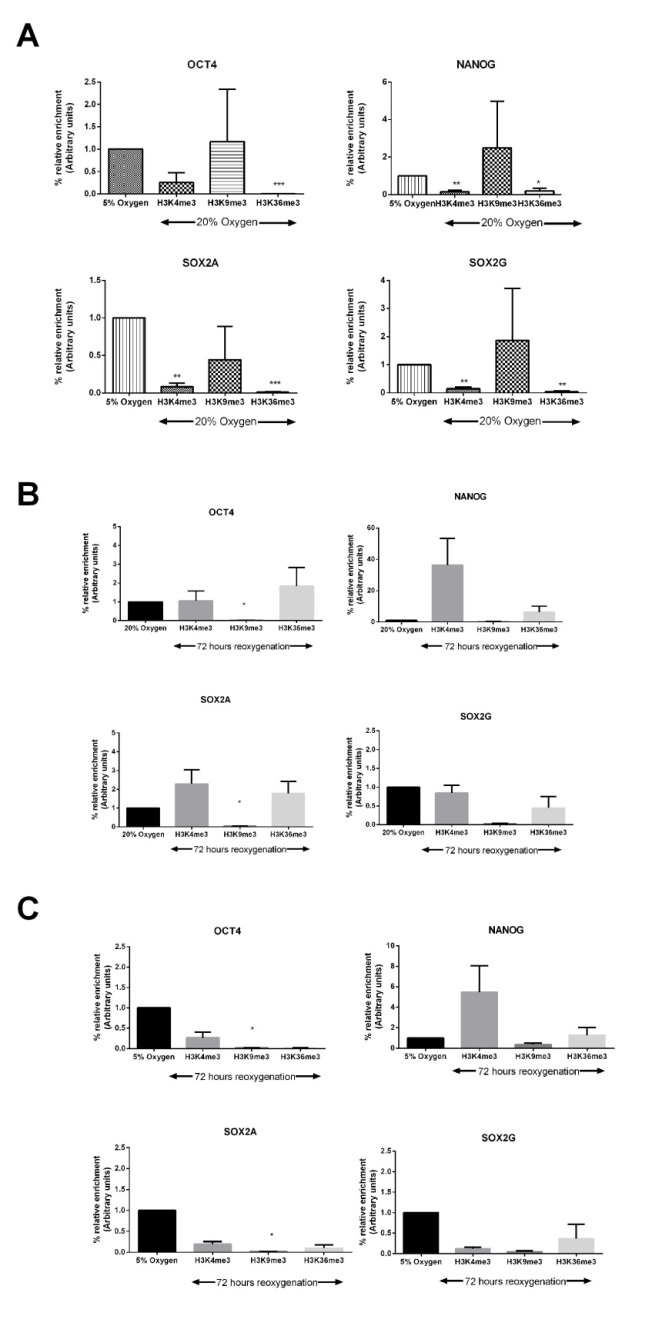


**Figure S2**

**Figure S3**

**Figure S4**

**Table S1**

| Gene |  | TaqMan Gene expression Assay |
| --- | --- | --- |
| OCT4 |  | Hs 01895061_u1 |
| SOX2 |  | Hs 00602736_s1 |
| NANOG |  | Hs 02387400_g1 |
| UBC |  | Hs 00824723_m1 |
| HIF-2α |  | Hs 01026142_m1 |

**Table S2**

| **OCT4 CR3** | **Sequence (5’→ 3’)** |
| --- | --- |
| Forward | TGAGAAGCCTTACTTAAGTCGACAGA |
| Reverse | TCAGCGTGCCCAGTC |
| Probe | TTCGAAGCTGTGGGGAGC |
| **NANOG** |  |
| Forward | TGGAAACGTGGTGAACCTAGAA |
| Reverse | AACCGAGCAACAGAACCTGAA |
| Probe | TATTTGTTGCTGGGTTTGT |
| **SOX2 G** |  |
| Forward | CGGCCACCACAATGGAAA |
| Reverse | TCCCTCCCACGCAGAGTTC |
| Probe | AGGCTGGTTCTGCT |
| **SOX2 A** |  |
| Forward | AACGGACGTGCTGCCATT |
| Reverse | TGTCCCGACGTAAAGATTTCAA |
| Probe | CCCTCCGCATTGAG |
| **FOXP3** |  |
| Forward | CCCCAGAGACCCTCAAATATCC |
| Reverse | CCCGAGGCAGGCAGAGA |
| Probe | CTCACTCACAGAATGGT |

**Table S3**

| **NANOG oct-sox element** |  |
| --- | --- |
| Forward | CGGTTTTCTAGTTCCCCACCTA |
| Reverse | CCAAGGCCATTGTAATGCAA |
| Probe | TCTGGGTTACTCTGCAGCT |
| **OCT4 oct-sox element** |  |
| Forward | GCCGTCTTCTTGGCAGACA |
| Reverse | CCCCAGGACAGAACCATCAC |
| Probe | AGAGAGATGCATGACAAAG |
| **SOX2 oct-sox element** |  |
| Forward | GGCCAGCCATTGTAATGCATAT |
| Reverse | GAGCAAGAACTGGCGAATGTG |
| Probe | CGGATTATTCACGTGGTAAT |
| **NANOG intermediated** |  |
| Forward | GGGTTTGTCTTCAGGTTCTGTTG |
| Reverse | GCTGCAGAGTAACCCAGACTAGGT |
| Probe | CGGTTTTCTAGTTCCC |

**Table S4**

| pGL3-NANOG Mut Forward | 5’-CTGATTTAAAAGTTGGAAACTCGAGGAA  CCTAGAAGTATTTGTTG-3’ |
| --- | --- |
| pGL3-NANOG Mut Reverse | 5’-CAACAAATACTTCTAGGTTCCTCGAGTTT  CCAACTTTTAAATCAG-3’ |
| oct-sox Mut BglII Forward | 5'-CTGCAGCTACTTTTGAGATCTAATGGCCTT  GGTGAG-3' |
| oct-sox Mut BglII Reverse | 5’-CTCACCAAGGCCATTAGATCTCAAAAGTAGC  TGCAG-3' |
